# Tetanus: The Use of Antitoxin

**Published:** 1897-08

**Authors:** C. Z. Laberge

**Affiliations:** Beaver Falls, Pa.


### TETANUS: THE USE OF ANTITOXIN.

By C. Z. Laberge, V.S.,
BEAVER FALLS, PA.

Bay draught-gelding, first seen May 5, 1897. Diagnosis: Tetanus; had been presenting symptoms of the disease for the last five days; deglutition impossible; urination and defecation *nil;* locomotion very difficult and painful; animal very much excited; temperature, 39.3° C. The horse was placed in an orchard, being the only place on the farm free from noise, and a mixture of chloroform, opium, and chloral, one ounce each, ordered. Mulford's tetanus antitoxin was obtained as soon as possible, and at 12 M. on the 6th 2 c.c. were injected, and the same quantity repeated at 8 P.M., with no perceptible change in the animal's condition. At 5 A.M. on the 7th the horse was somewhat better, having urinated, drank two bucketfuls of water, and eaten a quart of bran; temperature, 39.1° C.; no action of the bowels; 2 c.c. were given. At 2 P.M. the movements of the animal were less painful, breathing easier, and he ate a small portion of oats and was again injected with 2 c.c. of antitoxin. No action of the bowels having been obtained, 2 c.c. of eserine solution, 1 : 20, were given; temperature, 39° C. At 12 M. on the 8th temperature was 39° C.; movements of the animal were better; ate two quarts of oats, drinking freely, not so excited, kidneys working freely, no action of the bowels. Injected 15 grains of chloride of barium in 2 c.c. of water intravenously, followed in fifty-five minutes by a copious movement of the bowels and three others in the next fifteen minutes. May 9th the horse worse; temperature, 39.2° C.; animal more excited; not eating so well. The chloroform, opium, and chloral were continued, and on May 10th the horse was worse, temperature of over 40°, still able to eat and drink, but great difficulty in urinating and no action of the bowels. Gave another intravenous injection of 15 grains of chloride of barium and a little later 2 c.c. of tetanus antitoxin. At 11 P.M. the same day no change except animal quieter, and repeated the injection of 2 c.c. of antitoxin. May 11th, 7 A.M., temperature 39.2° C., eating well, urinates easily, but no action of the bowels. Renewed the injection of 15 grains of barium chloride intraveneously and had four good movements; later gave 2 c.c. of antitoxin. At 3 P.M. horse still better, very quiet; gave 2 c.c. antitoxin; temperature 39.2° C.; 11 P.M. temperature 39.1° C., walking easily, picking grass from the ground, drinking out of the trough. May 12th, horse continued to improve rapidly; temperature 39° C.; animal able to trot and run after his master for his feed, and chased the girls from the orchard, which was a very noticeable disposition of the horse when well. Animal kept on improving until May 27th, when he went to work, drawing stone from the quarries, going through the town, having to pass electric cars, but these in no way disturbed him.

**It will convey you through territory of our country that but few members traversed.—To Nashville, Sept. 7th, 8th, and 9th.**

**California may send here to this attractive point delegates to pave the way to the Pacific coast in 1899.**

Dr. Edward S. Kalb, of Rochester, Minnesota, reports a case of a horse injured by a collision on the race-track on June 2, 1896, producing an incision ten inches long on the right side, just below the cartilages of the false ribs, together with extensive bruises of the hind limbs, all of which he recovered from in about three weeks and went into his season's training in fair shape. On March 27, 1897, this horse received a purgation-ball consisting of one ounce of Barbadoes aloes, one drachm of calomel, and two drachms of ginger. It was followed by considerable griping and purgation in thirty-six hours. Patient continued in some pain, with a quick pulse and high temperature for one week, when he died.

Post-mortem revealed an adhesion of the cæcum to the abdominal wall, directly under the seat of injury, to which was attached an abscess involving the lower part of the cæcum and a portion of the large colon. The abscess had broken, but could be easily traced. The intestines were highly congested, and the peritoneum inflamed. The kidneys slightly hypertrophied and congested. Other organs normal.

The writer wishes to know which killed the horse, the injury or the pill?

